# Supplementary material for: Lactate sensing mechanisms in arterial chemoreceptor cells
Source: Nat Commun. 2021 Jul 6;12:4166. doi: 10.1038/s41467-021-24444-7 (PMC8260783; doi:10.1038/s41467-021-24444-7)
Supplement: Supplementary file 1 — Supplementary information [file 41467_2021_24444_MOESM1_ESM.pdf]

Supplementary Information

Lactate sensing mechanisms in arterial chemoreceptor cells

Hortensia Torres-Torrelo, Patricia Ortega-Sáenz, Lin Gao, and José López-Barneo

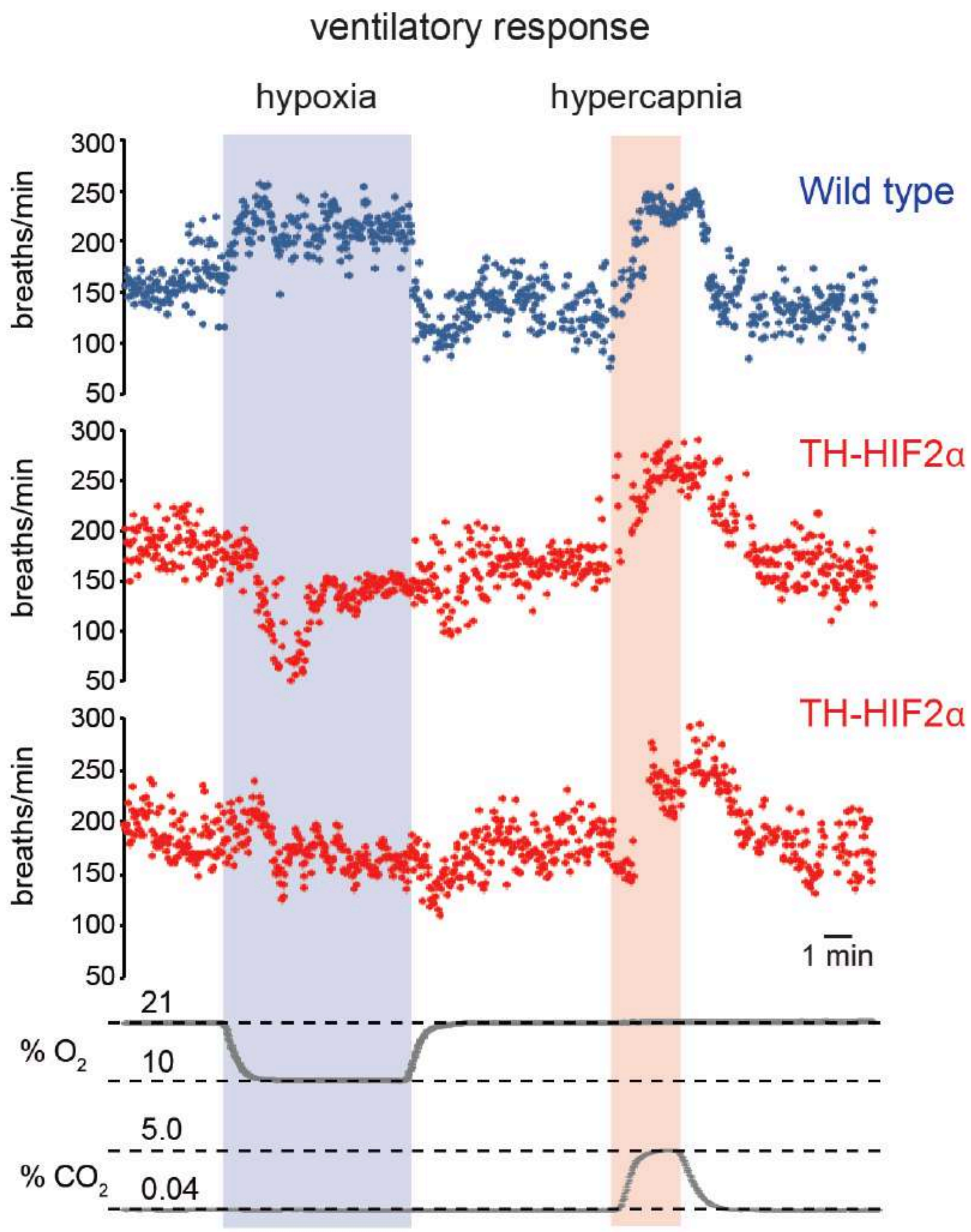

Supplementary Fig. 1. *Ventilatory response to hypoxia and hypercapnia of wild type and TH-HIF2α mice.* Representative examples of ventilatory responses (breaths/min) to hypoxia (10 % O_2, pale blue) and hypercapnia (5% CO_2, pale red) of wild type (blue dots) and TH-HIF2α (*Epas1* gene ablation in catecholaminergic cells) (red dots) mice. Note the abolition of the hypoxic ventilatory response in TH-HIF2α mice. Two examples of TH-HIF2α mice are shown to illustrate that in some cases (recording in the middle) exposure to hypoxia produced a transient apnea.

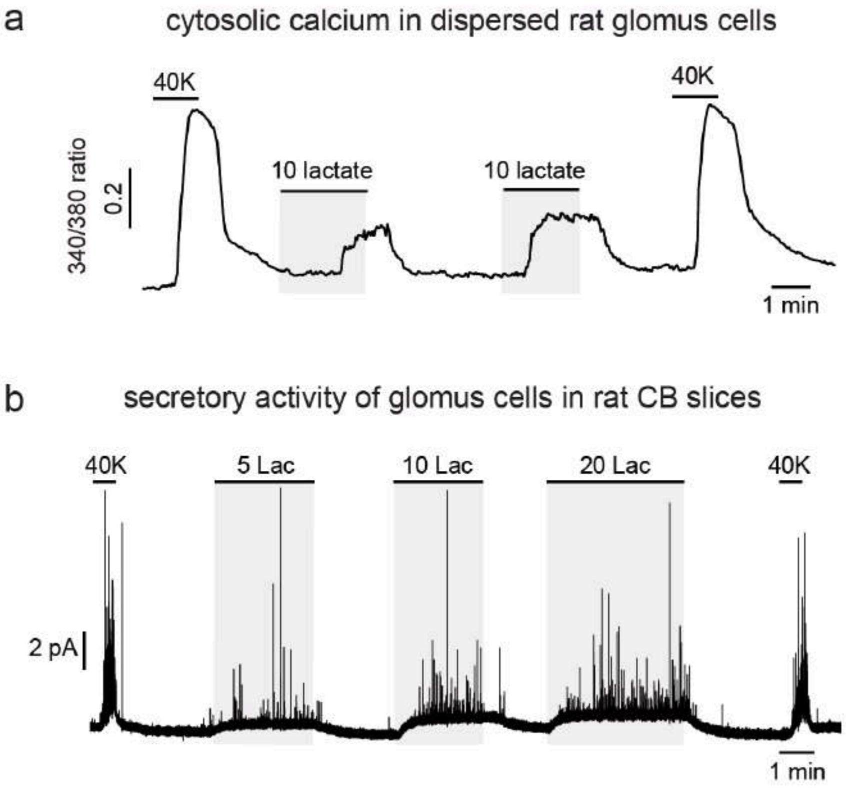

Supplementary Fig. 2. *Responses of rat glomus cells to lactate.* (**a**) Increase in cytosolic Ca^{2+} induced by K^+ (40 mM), and lactate (10 mM, grey) in a dispersed rat CB cell (14 similar recordings from 2 mice were performed). (**b)** Secretory glomus cell responses in a rat CB slice to high extracellular potassium (40K) and different lactate concentrations (in mM: 5, 10, 20; grey). (Representative recording of 7 similar experiments performed in 3 mice).

carotid body

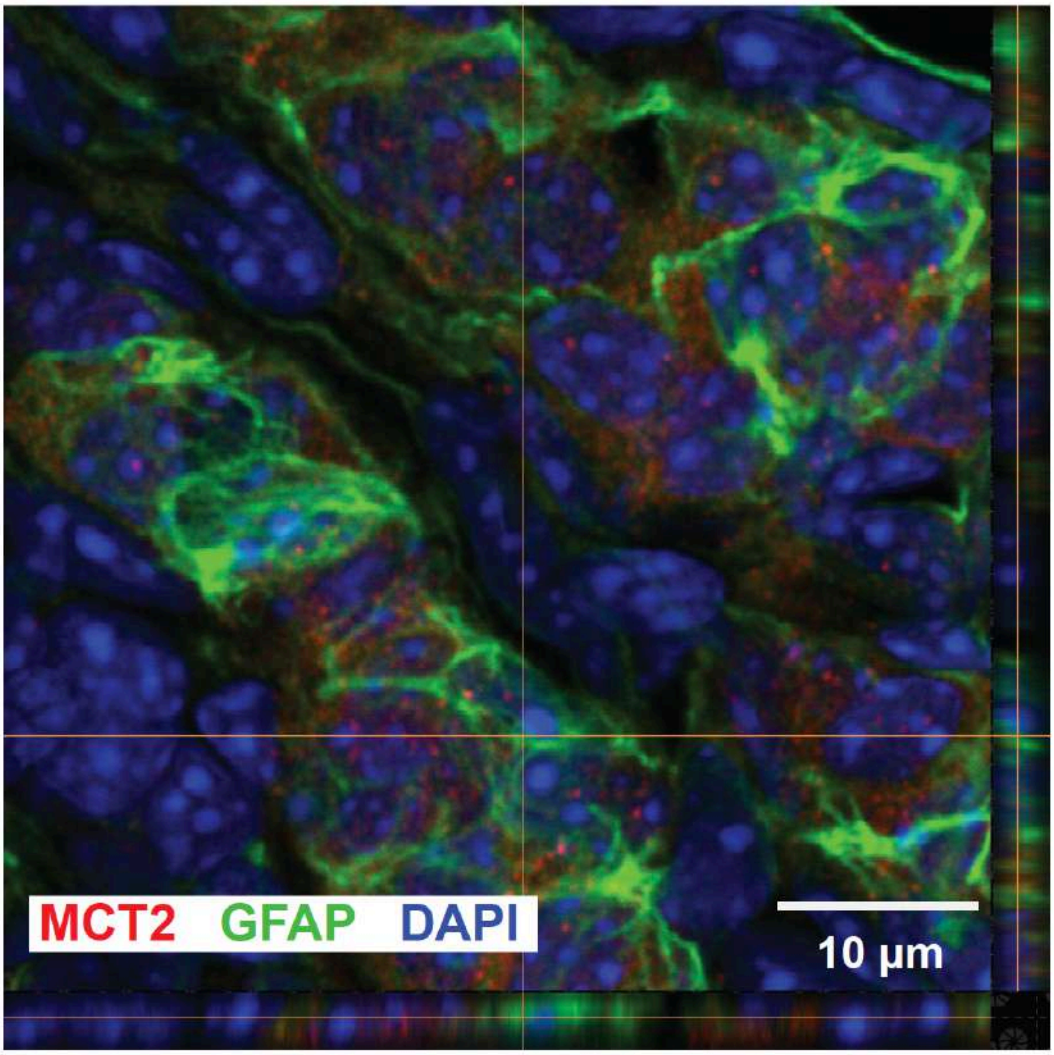

Supplementary Fig. 3. *Lack of expression of lactate transporter MCT2 in GFAP-positive carotid body cells.* Orthogonal projection from Z-stack confocal images of a carotid body histological section illustrating the lack of expression of MCT2 (red) in GFAP-positive cells (green). MCT2: monocarboxylate transporter 2. GFAP: glial fibrillary acidic protein. DAPI (dark blue) was used to stain nuclei. Similar data obtained in n=4 mice.

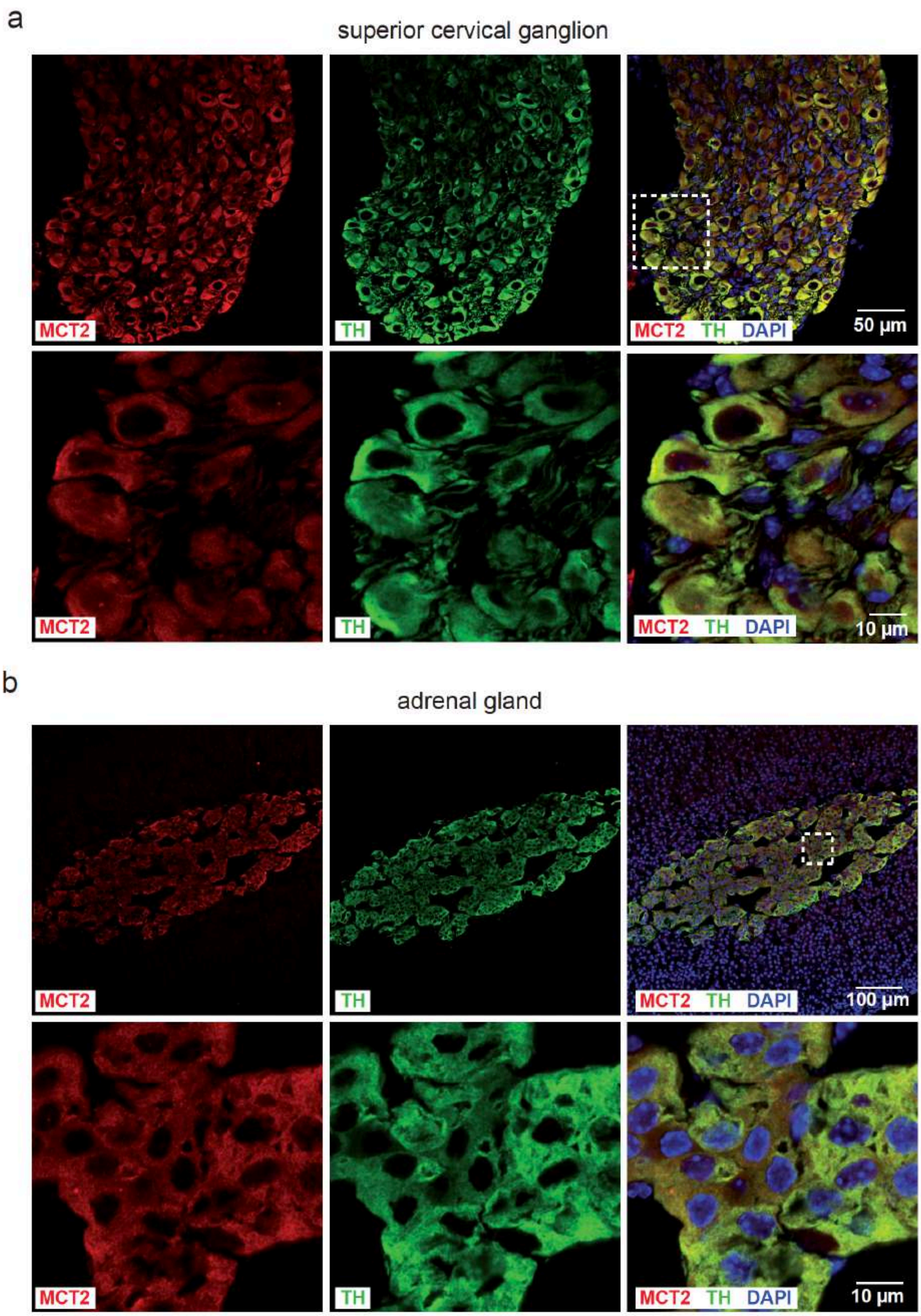

Supplementary Fig. 4. *Expression of lactate transporter MCT2 in neurons of the superior cervical ganglion and in adrenal medulla chromaffin cells.* (**a**) Top, Expression of MCT2 in superior cervical ganglion TH-positive neurons. Bottom, Magnification of the white dashed square indicated in the top panel. (**b)** Top, Colocalization of MCT2 in adrenal medulla TH-positive chromaffin cells. Bottom, magnification of the white dashed square indicated in the top panel. Note the lack of MCT2 expression in cells of the adrenal cortex. Similar data obtained in n=4 mice.

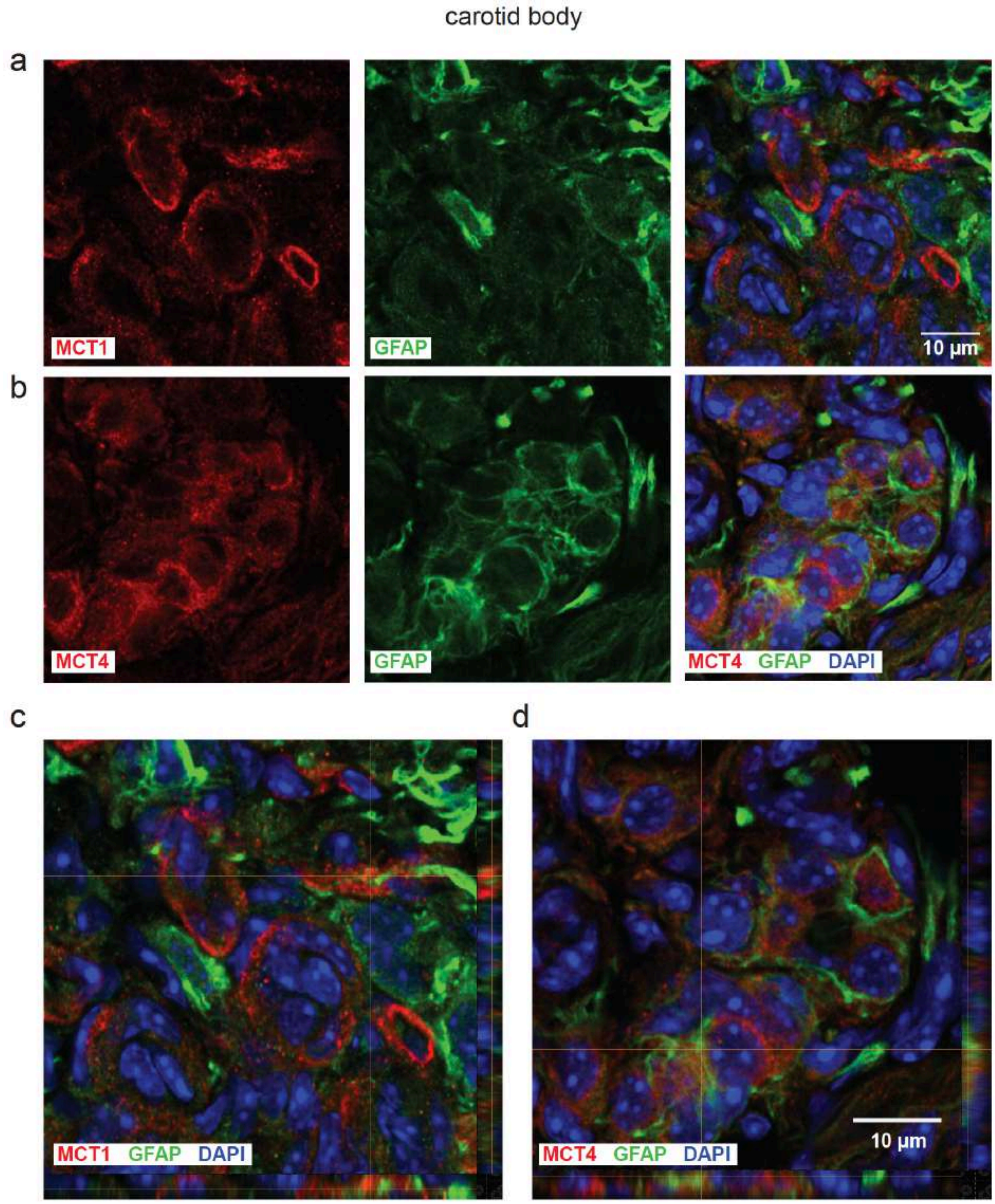

Supplementary Fig. 5. *Histological analysis of lactate transporters MCT1 an MCT4 in carotid body cells.* **(a)** Lack of expression of MCT1 in GFAP-positive carotid body cells. (**b**) Low level of expression of MCT4 in GFAP-positive carotid body cells. Scale bar for (**a**) and (**b**) is indicated in the upper right panel. (**c**) Orthogonal projection from Z-stack confocal images represented in (**a**). (**d**) Orthogonal projection from Z-stack confocal images presented in (**b**). MCT1: monocarboxylate transporter 1. MCT4: monocarboxylate transporter 4. GFAP: glial fibrillary acidic protein. DAPI (dark blue) was used to stain nuclei. Similar data obtained in n=4 mice.

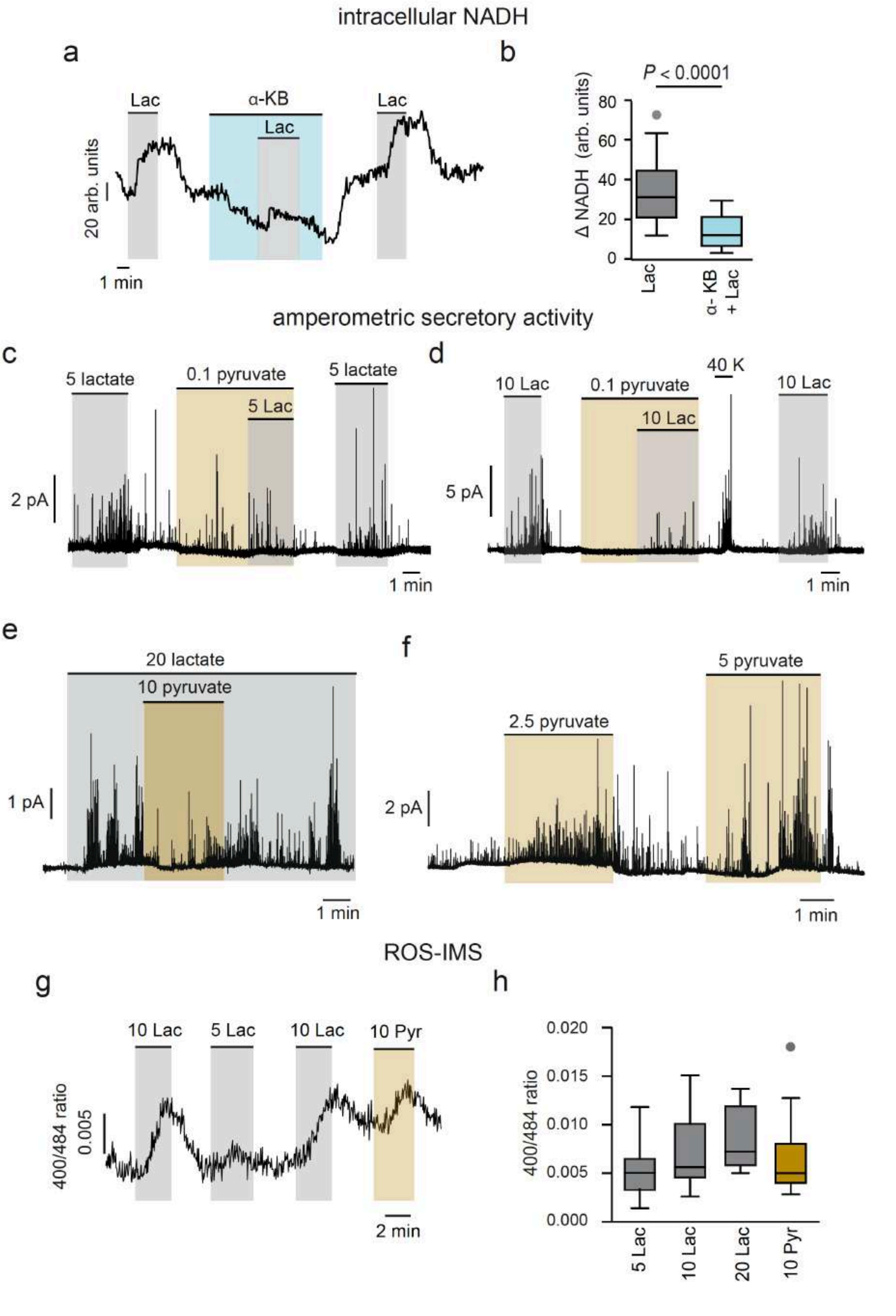

Supplementary Fig. 6. *Inhibition of lactate induced signals by pyruvate and α-ketobutyrate.* **(a)** Increase in NADH autofluorescence (ΔNADH) induced by lactate (Lac, 10 mM) in dispersed glomus cells and inhibition of the lactate response by α-

ketobutyrate (αKB, 2.5 mM; light blue). (**b**) Box plots representing data from 17 cells/2 mice. Mean values (in arbitrary units) are: 10 mM lactate: 33.8 ± 4.1; αKB and lactate (light blue): 14.8 ± 2.2. *P*-value calculated by paired, two-tails, t test. (**c, d**) Representative amperometric recordings from glomus cells in slices illustrating the inhibition of the secretory response induced by 5 mM (**c**) and 10 mM (**d**) lactate in the presence of 100 µM pyruvate. (**e**) Amperometric recording illustrating the secretory response of a glomus cell in a CB slice to lactate (20 mM) and inhibition by a pulse of pyruvate (10 mM). Note that the inhibitory effect of pyruvate is transient (7 similar recordings performed in 6 different mice). (**f**) Secretory responses of a glomus cell in a CB slice to pyruvate. Six similar recordings performed in 3 different mice. (**g**) Reversible increase in ROS at the glomus cells mitochondrial intermembrane space (IMS) induced by lactate (Lac, mM) and pyruvate (Pyr, mM). (**h**) Box plots representing the changes in ROS at the IMS induced by lactate (Lac, in mM) and pyruvate (Pyr, in mM). Calculated mean ± SEM values are: 5 mM lactate: 0.005 ± 0.0004, n=29; 10 mM lactate: 0.007 ± 0.0006, n=30; 20 mM lactate: 0.008 ± 0.001, n=9; 10 mM pyruvate: 0.006 ± 0.001, n=17 (5 mice). Exposures to lactate are shadowed in grey and exposures to pyruvate are shadowed in light brown. In the boxplots (panels b and h) the middle line is the median, the lower and upper hinges (IQR, interquartile range) correspond to the first and third quartiles, the upper whisker extends from the hinge to the largest value no further than 1.5 × IQR and the lower whisker extends from the hinge to the smallest value at most 1.5 × IQR. Data beyond the end of the whiskers (outliers) are plotted individually (grey). Arbitrary units (arb. units). Source data are provided as a Source Data file.

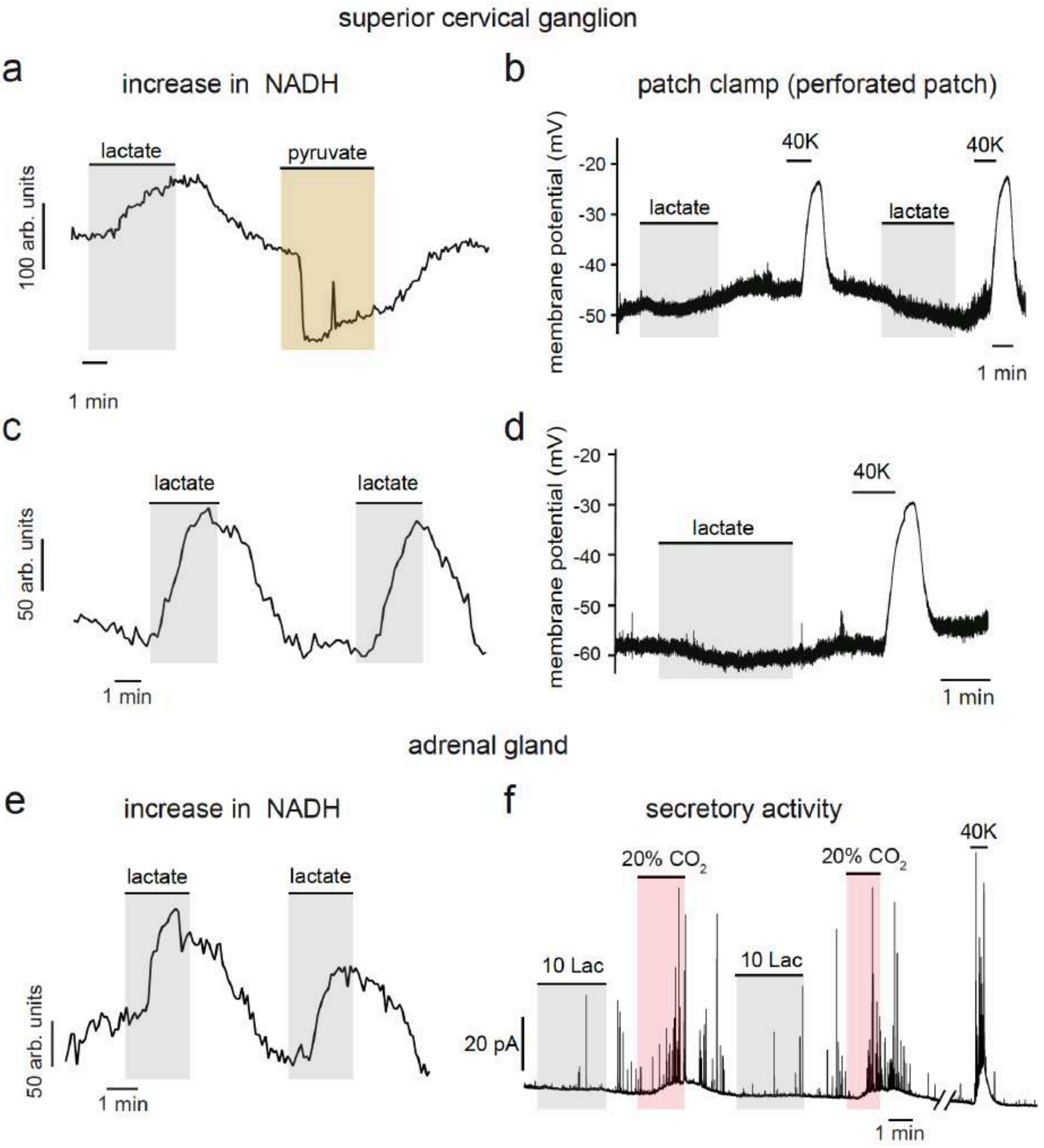

Supplementary Fig. 7. *Effects of lactate on superior cervical ganglion neurons and adrenal chromaffin cells.* (**a-d**) Representative changes (13 recordings/2 mice) in intracellular NADH induced by lactate (10 mM, grey) and pyruvate (10 mM, light brown) in dispersed neurons of the superior cervical ganglion (**a,c**). Perforated patch-clamp recordings (5 recordings/3 mice) obtained from the same cells (**b,d**) showed the lack of effect of lactate (grey) on the membrane potential of these cells, which were reversibly depolarized in response to high extracellular K^+. Note that action potentials in these neurons were filtered due to the low sampling interval used (4 ms) and appeared as a background "noise" which disappeared when cells were depolarized by high K^+ and Na^+ channels were inactivated. (**e**) Increase in NADH levels induced by 10 mM lactate (grey) in an isolated adrenal medulla chromaffin cell (10 similar recordings/2mice). (**f**) Representative amperometric recording of the secretory activity induced by hypercapnia (20% CO_2, pale red) 10 mM lactate (grey) and high potassium on chromaffin cells in adrenal slices of adult mice (10 similar recordings performed in 3 different animals). Arbitrary units (arb. units).

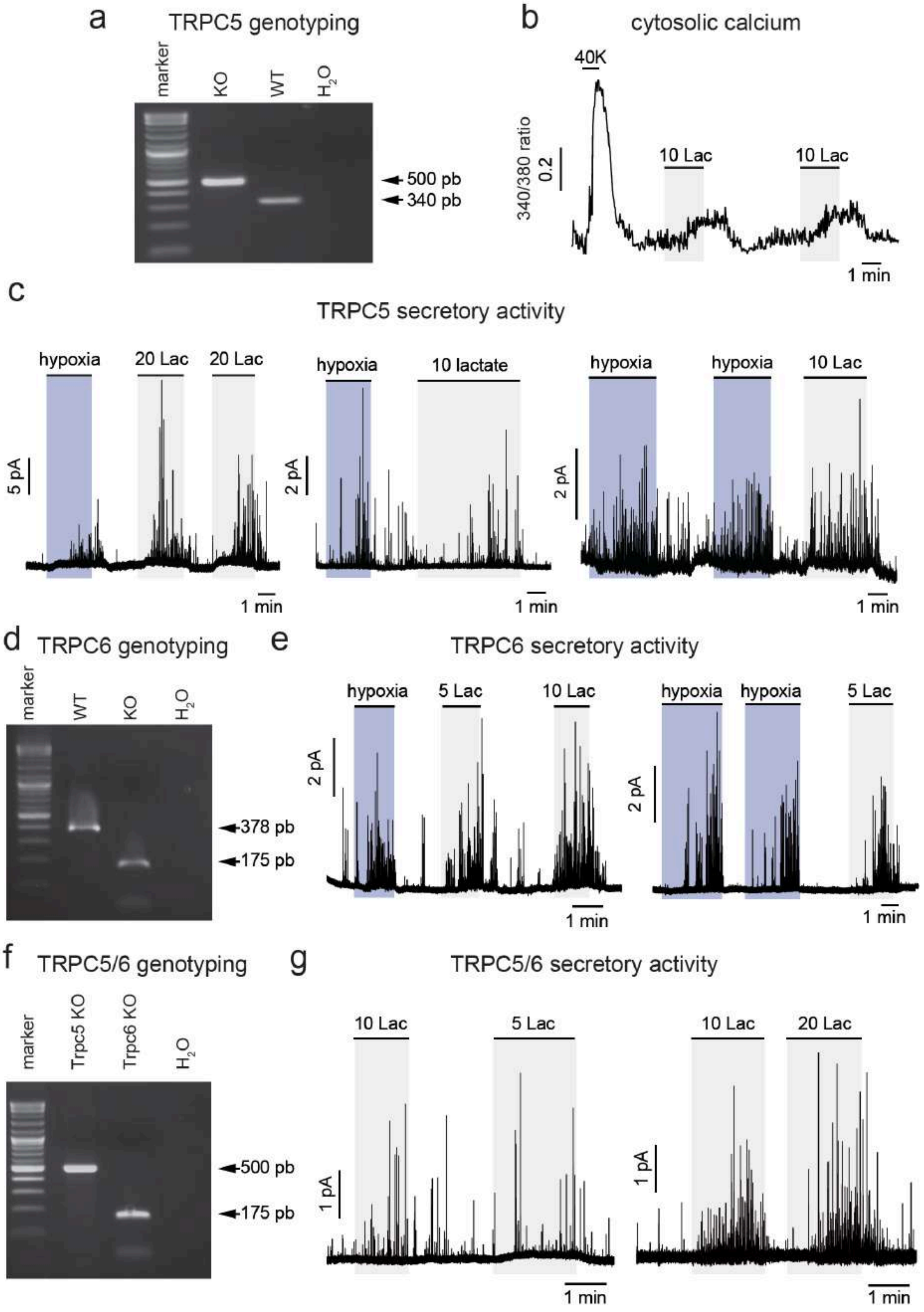

Supplementary Fig. 8. *Lactate-induced activation of glomus cells from mice lacking TRPC5 and TRPC6 channels***.** (**a**) Genotyping of control (WT) and TRPC5 knockout (KO) mice. (**b**) Increase in cytosolic Ca^{2+} induced by K^{+} (40 mM), and lactate (10 mM) in a TRPC5-deficient dispersed glomus cell (10 similar recordings from 2 mice). (**c**)

Examples of secretory responses to hypoxia (O_2 tension ≈15 mm Hg) and lactate of three different TRPC5-deficient glomus cells (6 similar recordings from 3 mice). (**d**) Genotyping of control (WT) and TRPC6 knockout (KO) mice. (**e**) Separate examples of secretory responses to hypoxia (O_2 tension ≈15 mm Hg) and lactate (5 and 10 mM) of two different TRPC6-deficient glomus cells (5 similar recordings were performed in 3 mice) (**f**) Genotyping of control (WT) and TRPC5/6 double knockout (KO) mice. (**e**) Separate examples of secretory responses to lactate (5, 10 and 20 mM) of two different TRPC5/6-deficient glomus cells (8 similar recordings in 2 mice). Exposures to lactate are shadowed in grey and exposures to hypoxia are shadowed in light blue. Uncropped and unprocessed scans of the gels are provided in the Source Data file.

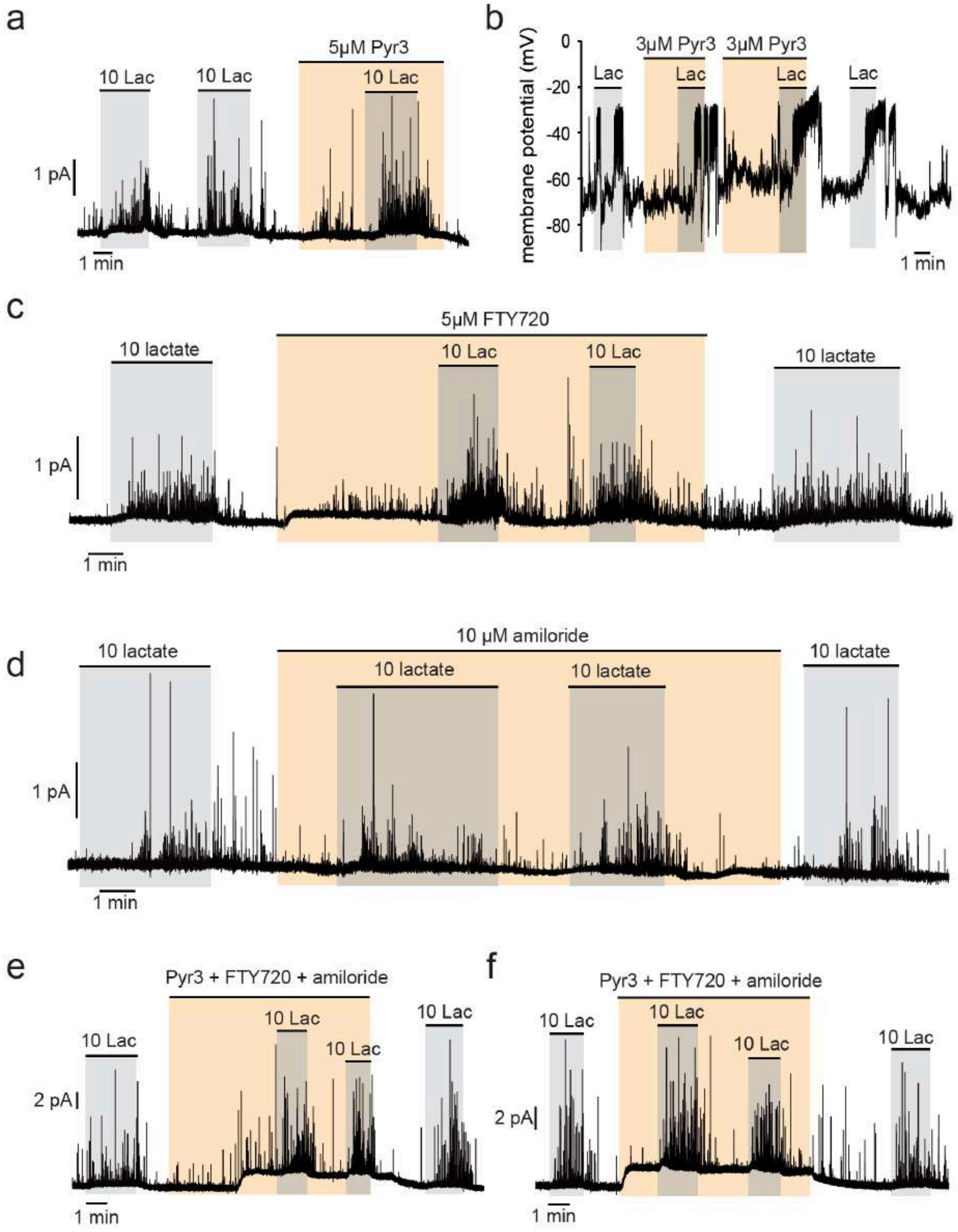

Supplementary Fig. 9. *Effect of pharmacological blockade of cationic channels on glomus cell activation by lactate.* (**a**) Representative amperometric recording showing the lack of effect of 5 μM Pyr3 (a blocker of TRP3 channels) on the secretory activity induced by 10 mM lactate in glomus cells (3 similar recordings performed in 2 mice). (b) Lack of effect of 3 μM Pyr3 on lactate (10 mM)-induced glomus cell membrane depolarization. (**c**) Representative amperometric recording showing the lack of effect

of 5 μM FTY720 (a blocker of TRPM7 channels) on the secretory activity induced by 10 mM lactate in glomus cells (3 similar recordings performed in 3 mice). (**d**) Amperometric recording showing the lack of effect of 10 μM amiloride (a blocker of epithelial Na^+ channels) on the secretory activity induced by 10 mM lactate in glomus cells (2 similar recordings performed with amperometry in 2 different cultures). (**e, f**) Amperometric recordings showing the lack of effect of Pyr3, FTY720 and amiloride on the lactate secretory response in glomus cells (2 similar recordings performed in 2 different cultures). Exposures to lactate are shadowed in grey and exposures to the different ion channels blockers are shadowed in light orange.

	name	sequence
trpc5+ (WT)	35594	5’-CATCAGTGTTTCTTGCTGCAC-3’
	35595	5’-GTAGCCCCCTTTCGACTTTC-3’
trpc5-(KO)	35594	5’-CATCAGTGTTTCTTGCTGCAC-3’
	35596	5’-GCATACTCTTGGGCTCTTTTCA-3’
trpc6+ (WT)	20556	5´-TCTTTATGCAATCGCTGTGG-3´
	20557	5´-GCTAGTCT-TCCTGCAATCCA-3´
trpc6-(KO)	20635	5´-TCTATTAACACTCAACTGGCACCT-3´
	oIMR7415	5´-GCCAGAGGCCACTTGTGTAG-3´

Supplementary Table 1.
