# Supplementary material for: Lactate sensing mechanisms in arterial chemoreceptor cells
Source: Nat Commun. 2021 Jul 6;12:4166. doi: 10.1038/s41467-021-24444-7 (PMC8260783; doi:10.1038/s41467-021-24444-7)
Supplement: Supplementary file 3 — Reporting summary [file 41467_2021_24444_MOESM3_ESM.pdf]

nature research

Corresponding author(s): DBPR, Ms. NCOMMS-20-29211B

Last updated by author(s): May 25, 2021

Reporting Summary

Nature Research wishes to improve the reproducibility of the work that we publish. This form provides structure for consistency and transparency in reporting. For further information on Nature Research policies, see our Editorial Policies and the Editorial Policy Checklist.

Statistics

For all statistical analyses, confirm that the following items are present in the figure legend, table legend, main text, or Methods section.

n/a	Confirmed	
☐	☒	The exact sample size (n) for each experimental group/condition, given as a discrete number and unit of measurement
☐	☒	A statement on whether measurements were taken from distinct samples or whether the same sample was measured repeatedly
☐	☒	The statistical test(s) used AND whether they are one- or two-sided<br>*Only common tests should be described solely by name; describe more complex techniques in the Methods section.*
☒	☐	A description of all covariates tested
☐	☒	A description of any assumptions or corrections, such as tests of normality and adjustment for multiple comparisons
☐	☒	A full description of the statistical parameters including central tendency (e.g. means) or other basic estimates (e.g. regression coefficient) AND variation (e.g. standard deviation) or associated estimates of uncertainty (e.g. confidence intervals)
☐	☒	For null hypothesis testing, the test statistic (e.g. F, t, r) with confidence intervals, effect sizes, degrees of freedom and P value noted<br>*Give P values as exact values whenever suitable.*
☒	☐	For Bayesian analysis, information on the choice of priors and Markov chain Monte Carlo settings
☒	☐	For hierarchical and complex designs, identification of the appropriate level for tests and full reporting of outcomes
☒	☐	Estimates of effect sizes (e.g. Cohen's d, Pearson's r), indicating how they were calculated

Our web collection on statistics for biologists contains articles on many of the points above.

Software and code

Policy information about availability of computer code

Data collection	All the commercial software used for data collection is clearly described in Methods and below (Data Analysis)
Data analysis	Pulse-Pulsefit version 8.80 from HEKA GmbH<br>Igor Pro Folder version 4.08 Carbon from WaveMetrics, Inc.<br>Aquacosmos version 2.64 Release 2.6.4.0 from Hamamatsu Photonics<br>Iox version 2 from Emka Technologies<br>Prism Version 8.2.1 (279) for MacOS

For manuscripts utilizing custom algorithms or software that are central to the research but not yet described in published literature, software must be made available to editors and reviewers. We strongly encourage code deposition in a community repository (e.g. GitHub). See the Nature Research guidelines for submitting code & software for further information.

Data

Policy information about availability of data

All manuscripts must include a data availability statement. This statement should provide the following information, where applicable:

- Accession codes, unique identifiers, or web links for publicly available datasets
- A list of figures that have associated raw data
- A description of any restrictions on data availability

A data availability statement has been included in the text:
Data available on request from the authors (The data that support the findings of this study are available from the corresponding author upon reasonable request).
Authors can confirm that all relevant data are included in the paper and/ or its supplementary information files. The authors declare that [the/all other] data

supporting the findings of this study are available within the paper [and its supplementary information files]

Field-specific reporting

Please select the one below that is the best fit for your research. If you are not sure, read the appropriate sections before making your selection.

[x] Life sciences   [ ] Behavioural & social sciences   [ ] Ecological, evolutionary & environmental sciences

For a reference copy of the document with all sections, see nature.com/documents/nr-reporting-summary-flat.pdf

Life sciences study design

All studies must disclose on these points even when the disclosure is negative.

Sample size	We defined the number of independent measurements necessary to ascertain if two parameters have similar or different values based on the experimental evidence and the previous experience in our laboratory.
Data exclusions	No data were excluded from the analyses
Replication	The data obtained from in vivo (lactate meassurements and pletismography) and in vitro preparations were systematically and clearly replicated in several independent experiments performed in different days (animals or in vitro cultures) as stated in the figure legends
Randomization	Most of the in vivo (plethismography) and in vitro (amperometry, patch-clamp and microfluorimetry) experiments were performed on WT animals (and cells) with a mixed genetic background provided by the IBiS's animal facility. Animals used were between 1 and 3 months of age and without sex selection. For experiments on genetically modified animals (in vivo plethysmography and in vitro recordings), mice were used in a ramdomized way, although a WT and a KO mice of the same sex were studied in parallel in each experiment to facilitate comparison. When recording and analysis were completed, the genotype was confirmed.<br>The study of covariates does not apply to our experiments. In "in vitro" experiments we performed recordings from a given cell (control data) and afterwards applied extracellulary lactate, pyruvate, hypoxia, ion channels blockers, etc to the same cell to check for the experimental effects. In practically all data used in this paper reversibility of the effects was a necessary condition to be included in the analysis.
Blinding	In case of experiments conducted in genetically modified animals, whenever possible, all the in vivo (plethismography) and in vitro (amperometry and patch-clamp and microfluorimetry) experiments were carried out blind, taking note of the number of the animal and always including a WT and a KO for comparison (see above). When recording and analysis were completed, the genotype was revealed to be included in the corresponding group.<br>Whenever possible, the selection of the WT and KO mice used for an experiment was done by a person (normally a person working in the animal facility) who was not going to carry out the experiments. Only when no one was available, the same person conducting the experiments picked up the mice from the animal facility, although animals were transported to the lab in identical cages and genotype of the mice was not confirmed until the end of the experiment.

Reporting for specific materials, systems and methods

We require information from authors about some types of materials, experimental systems and methods used in many studies. Here, indicate whether each material, system or method listed is relevant to your study. If you are not sure if a list item applies to your research, read the appropriate section before selecting a response.

Materials & experimental systems

n/a	Involved in the study	
[ ]	[x]	Antibodies
[x]	[ ]	Eukaryotic cell lines
[x]	[ ]	Palaeontology and archaeology
[ ]	[x]	Animals and other organisms
[x]	[ ]	Human research participants
[x]	[ ]	Clinical data
[x]	[ ]	Dual use research of concern

Methods

n/a	Involved in the study	
[x]	[ ]	ChIP-seq
[x]	[ ]	Flow cytometry
[x]	[ ]	MRI-based neuroimaging

Antibodies

Antibodies used	Primary antibodies:<br>For MCT1: 1ª Ab (rabbit, Novus Biologicals, Cat. No. NBP1-59656, 1:200 dilution)<br>For MCT2: 1ª Ab (rabbit, Thermofisher Scientific, Cat. No. PA5-77498, 1:500 dilution)<br>For MCT4: 1ª Ab (rabbit, Proteintech, Cat. No. 22787-1-AP, 1:100 dilution),<br>For TH: 1ª Ab (sheep,Millipore/Merck, Cat. No. AB1542, 1:200 dilution)<br>For GFAP: 1ª Ab (chicken, Abcam, Cat. No. ab4674, 1:500 dilution)<br>2ª Ab: Goat-anti-rabbit Alexa Fluor 568 (Thermofisher Scientific, Cat. No. A11011, 1:400 dilution)

	2ª Ab: Goat anti-chicken Alexa Fluor 488 (Jackson ImmunoResearch, Cat. No. 103-545-155, 1:400 dilution)<br>2ª Ab: Donkey anti-sheep Alexa Fluor 488 (Thermofisher Scientific, Cat. No. A11015, 1:400 dilution)
Validation	Mouse TH primary antibody has previously been used in our laboratory (see Gao et al., 2017; 595.18 pp 6091–6120).<br>MCT1, was validated in submucosa of mouse intestine (see https://www.novusbio.com).<br>MCT2 was validated for Immunohistochemical analysis, in perfusion-fixed, frozen mouse brain (see https://www.thermofisher.com/es/es/home.html).<br>MCT4 was validated in fibroblast (Pub Med ID 33262836).<br>GFAP primary antibody was validated in mouse hippocampus free-floating sections (see https://www.abcam.com/)

Animals and other organisms

Policy information about studies involving animals; ARRIVE guidelines recommended for reporting animal research

Laboratory animals	TH-NDUFS2 (B6/129SV background) Laboratory of José López-Barneo. (Fernandez Aguera et al., 2015)<br>TH-HIF2a (129Sv:C57BL/6 background) Laboratory of José López-Barneo. (Macías et al; 2018)<br>ERT2-HIF2a (129Sv:C57BL/6 background)Laboratory of José López-Barneo. (Moreno-Dominguez et al., 2020)<br>TRPC5 (The Jackson Laboratory; complete mouse line name: 129S1/SvImJ-Trpc5tm1.1Clph/J; Stock No: 030804; Strain: 129S1)<br>TRPC6 (The Jackson Laboratory; complete mouse line name: B6J;129S8-Trpc6tm1Lbi/Mmjax)<br>Double TRPC5/6 knockout mice were generated in our animal facility (Instituto de Biomedicina de Sevilla).<br>Animals 1-3 months old of both sexes were used in this study. For adult conditional KOs, mice 4-5 months old were used without selecting animal sex. Mice were housed in a controlled environment with a 12-h light-dark cycle, 20-24ºC ambient temperature and without exceeding 55% humidity.
Wild animals	No wild animals were used in the study
Field-collected samples	The study did not involve samples collected from the field
Ethics oversight	All procedures were approved by the Institutional Committee of the University of Seville for Animal Care and Use (2012PI/LB02 and 22-09-15-332). Handling of the animals was conducted in accordance with the European Community Council directives 86/609/EEC, and 2010/63/EU for the Care and Use of Laboratory Animals.

Note that full information on the approval of the study protocol must also be provided in the manuscript.
